# Swin-MFA: A Multi-Modal Fusion Attention Network Based on Swin-Transformer for Low-Light Image Human Segmentation

**DOI:** 10.3390/s22166229

**Published:** 2022-08-19

**Authors:** Xunpeng Yi, Haonan Zhang, Yibo Wang, Shujiang Guo, Jingyi Wu, Cien Fan

**Affiliations:** Electronic Information School, Wuhan University, Wuhan 430064, China

**Keywords:** multi-modal fusion network, segmentation, low light environment, depth-sensing

## Abstract

In recent years, image segmentation based on deep learning has been widely used in medical imaging, automatic driving, monitoring and security. In the fields of monitoring and security, the specific location of a person is detected by image segmentation, and it is segmented from the background to analyze the specific actions of the person. However, in low-illumination conditions, it is a great challenge to the traditional image-segmentation algorithms. Unfortunately, a scene with low light or even no light at night is often encountered in monitoring and security. Given this background, this paper proposes a multi-modal fusion network based on the encoder and decoder structure. The encoder, which contains a two-branch swin-transformer backbone instead of the traditional convolutional neural network, fuses the RGB and depth features with a multiscale fusion attention block. The decoder is also made up of the swin-transformer backbone and is finally connected via the encoder with several residual connections, which are proven to be beneficial in improving the accuracy of the network. Furthermore, this paper first proposes the low light–human segmentation (LLHS) dataset of portrait segmentation, with aligned depth and RGB images with fine annotation under low illuminance, by combining the traditional monocular camera and a depth camera with active structured light. The network is also tested in different levels of illumination. Experimental results show that the proposed network has good robustness in the scene of human segmentation in a low-light environment with varying illumination. The mean Intersection over Union (mIoU), which is often used to evaluate the performance of image segmentation model, of the Swin-MFA in the LLHS dataset is 81.0, is better than those of ACNet, 3DGNN, ESANet, RedNet and RFNet at the same level of depth in a mixed multi-modal network and is far ahead of the segmentation algorithm that only uses RGB features, so it has important practical significance.

## 1. Introduction

Image segmentation is an important subject in the field of computer vision, the purpose of which is to segment specific objects from various backgrounds [1,2]. The edge, color, texture and many other features of the image are used as the standard to segment the image into different regions by the traditional methods. For example, based on threshold [3], edge [4] and clustering [5,6], these traditional segmentation methods are relatively simple but cannot segment images accurately in complex scenes. Therefore, image segmentation based on deep learning with a higher accuracy has become a research hotspot.

Starting from the proposal of a fully convolutional neural network (FCN) [7], semantic segmentation algorithms based on neural networks have appeared on the stage. An FCN extends the image classification task to the image segmentation task with the pixel level, which lays a foundation for the current image semantic segmentation research. At present, in order to enhance the effect of semantic segmentation, there is more and more research on new semantic segmentation based on neural networks.

With the improvement of the efficiency and accuracy of image segmentation, image segmentation, especially human segmentation, has shown a wide application prospect in many fields. However, in order to ensure safety and reliability in the fields for automobile navigation and security monitoring, image segmentation is often required to have the ability to process the images of various scenes during the whole day and even at night. In a night scene, the RGB image often has uneven illumination, low light intensity or even no light at all. An image segmentation algorithm only based on the RGB feature usually cannot work well in this situation. Therefore, many recent studies have also carried out attempts to solve this problem, including using mixed datasets of day and night for adversarial training [8] and the introduction of thermal images for multi-modal fusion [9]. However, these studies are all passive imaging, still subject to environmental constraints. The RGB-D cameras that have been developed in recent years can collect the depth data of scenes through active structured light, which means they are less dependent on the environment itself, providing a new possibility for night-time image segmentation.

**Figure 1:**
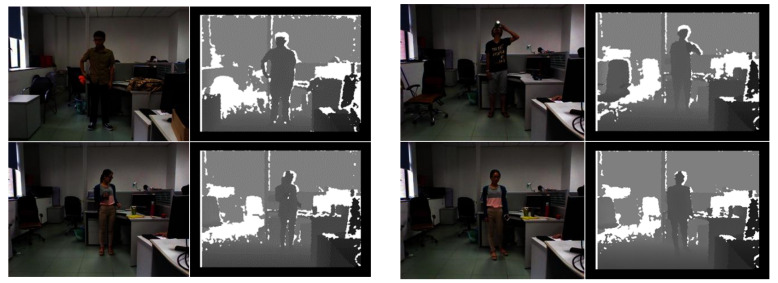
The existing RGB-D datasets focused on human segmentation.

At present, research on RGB-D image segmentation mainly focuses on solving the fusion problem of the RGB image and depth image and the inaccurate measurement problem of depth images [10], while little research notices its potential application in low-illumination scenes. Therefore, this paper conducts research on image segmentation at night and proposes a transformer-based neural network and feature-fusion attention mechanism. The self-attention mechanism is used to replace a traditional convolutional neural network (CNN), with the purpose of realizing compensation for the loss of information in RGB images with depth information and, finally, achieve the goal of image segmentation in low-illumination scenes. Notice that the existing RGB-D datasets, such as SUN RGB-D, Cityscapes and NYU Depth V2, are not dedicated datasets for low-illuminance conditions as shown in Figure 1. In addition, unfortunately, the existing low-light datasets have either a single background or poor quality. This paper introduces a human segmentation dataset in low light scenes.

## 2. Relative Work

At present, single-mode image segmentation has made great progress in segmentation accuracy and efficiency. In ref. [11], U-net uses the short channels to splice encoding and decoding parts, which retain more original information, solving the problem of gradient disappearance to some extent. In ref. [12], ICnet, which uses multi-scale images as input and a cascaded network to improve efficiency, was proposed. Moreover, ICnet limits the input size of images by scaling, thus increasing the reasoning speed of the network.

A transformer was initially applied in natural language processing (NLP) [13]. Recently, many works have transplanted the transformer to CV tasks and achieved good results [14]. In ref. [15], a Vision Transformer was proposed, which cut the serialized data of images into small pieces as the input of the transformer, demonstrating the powerful capability of a transformer in the field of computer vision. In ref. [16], a Vision Transformer using shifted window was proposed, which has strong performance in image classification. Moreover, a Dense-Transformer was proposed to capture the sequence spectral relationship in ref. [17], realizing hyperspectral image (HSI) classification. Some reinforced transformers, such as RTN [18], were used for the automatic quality evaluation of medical images. Transformers were used in the image segmentation in the earlier period in ref. [19], where the transformer was used to completely replace the encoding part of a traditional FCN with the attention mechanism. However, its application in multi-mode and multi-feature was still lacking. Moreover, the transformer’s performance in variable lighting and low-lighting conditions remains to be seen.

At present, there has been much research on RGB-D image segmentation, but little research on RGB-D complementarity in low-illumination conditions. Moreover, some RGB-D datasets are of low quality and without fine annotation. In ref. [20], NIN network was used to extract depth image features and integrate them into the GoogLeNet network. In ref. [21], the LDFNet was proposed, which incorporates luminance, depth and color information by a fusion network. In ref. [22], a 3D graph neural network (3DGNN) was proposed to construct a k-nearest neighbor graph based on a KNN pair 3D-point-cloud graph. In ref. [23], three ResNet network branches are adopted, in which two branches are used to extract RGB and depth image features, and the other branch is used to fuse RGB and depth image features. However, the relevant performance of network image segmentation at night and other complex scenes has not been studied, which needs further research and confirmation.

In short, in order to solve the problem that the existing image-segmentation methods cannot be applied to low illumination scenes, the contributions of this paper are as follows:

- Human body images are segmented in the multi-modal and multi-feature way in low-illumination scenes, by using the fusion information of the depth image and RGB image as the segmentation basis.
- A multi-modal end-to-end segmentation network based on swin-transformer is proposed, which realizes end-to-end RGB and depth feature-fusion attention by combining swin-transformer features that are demonstrated to be stable under changeable-lighting conditions. It can totally replace the traditional convolutional neural network and improve the accuracy of segmentation.
- Aiming at the shortcomings of traditional image segmentation under low illumination, a modified and pre-processed body semantic segmentation dataset (LLHS) with fine annotation for a low-light scene is proposed, which is much larger in scale and scene than the previous dataset, filling the gap in the semantic segmentation dataset in the low-illuminance condition.

## 3. Materials and Methods

Swin-MFA proposed in this paper is an end-to-end multi-modal segmentation network with low illumination optimization. This network adopts encoder and decoder structure with transformer backbone, which has good noise tolerance and accuracy for human segmentation in low light conditions. In addition, the dataset LLHS was produced for the deep multi-modal method to solve the problem of human body segmentation in low-illumination scenes, which has good advantages in terms of the size and quality.

### 3.1. Low Light Human Segmentation Dataset

Low light–human segmentation dataset is a new portrait dataset in low-light scenes, which adopts active ranging sensing method to collect depth images based on structured light principle and collect RGB images by a traditional RGB camera. Due to the black vacancy at the edge of the portrait in the depth image and the registration problem between the depth image and RGB image, the dataset is preprocessed as follows.

(1) The physical location of the camera of RGB image and depth image results in different spatial-coordinate systems. The images taken by RGB camera and depth camera are not matched by pixels, so it is necessary to register RGB image and depth image. The internal parameter matrix and external parameter matrix in different scenes are obtained by calibrating RGB camera and depth camera, respectively. Then, the transformation matrix of two coordinate systems is calculated by Equation (1):
  (1) $$p_{rgb}=H_{rgb}(RH_{ir}^{-1}p_{ir}+T)$$
  where, *p_ir_* is the coordinates of pixels in the depth image before processing, *H_ir_* is the internal parameter matrix of the depth camera and *H_rgb_* is the internal parameter matrix of the RGB camera. *R* and *T* are rotation matrices and shift vectors, respectively, derived from the outer parameter matrix.
  (2) $$R=R_{rgb}R_{ir}^{-1}$$
  (3) $$T=T_{rgb}-R_{rgb}R_{ir}^{-1}T_{ir}$$
  where $R_{ir}(R_{rgb})$ and $T_{ir}(T_{rgb})$ are rotation matrix and shift vector of depth camera (RGB camera) in external parameter matrix, respectively.
(2) In depth images, due to camera shooting angle and objects blocking, black gaps appear in the image, resulting in the interference of the image edge information, which needs to be processed. The depth camera of Realsense device is set on the left side, and the imaging algorithm is realized by referring to the left camera. Therefore, the upper and lower five pixels of the left side adjacent to the black gap can be used as the processing neighborhood to fill the vacancy. In order to maintain image-edge information, it is necessary to make the filled pixels contain background information rather than foreground information. Therefore, the pixel of the farthest point with the largest pixel value in the neighborhood is used to fill the black vacancy. The specific calculation formula can be expressed in Equation (4).
  (4) $${P^{\prime }}_{\left(i,j\right)}=MAX\{P_{\left(i-1,j\right)},P_{\left(i+1,j\right)},P_{\left(i,j-1\right)},P_{\left(i-1,j-1\right)},P_{\left(i-1,j+1\right)}\}$$
  where *P_(i,j)_* is the pixel value of the *i*-th row and *j*-th column in the filling kernel, and *P*’_(*i,j)*_ is the corresponding pixel value of the *i*-th row and *j*-th column in the image after processing.

**Figure 2:**
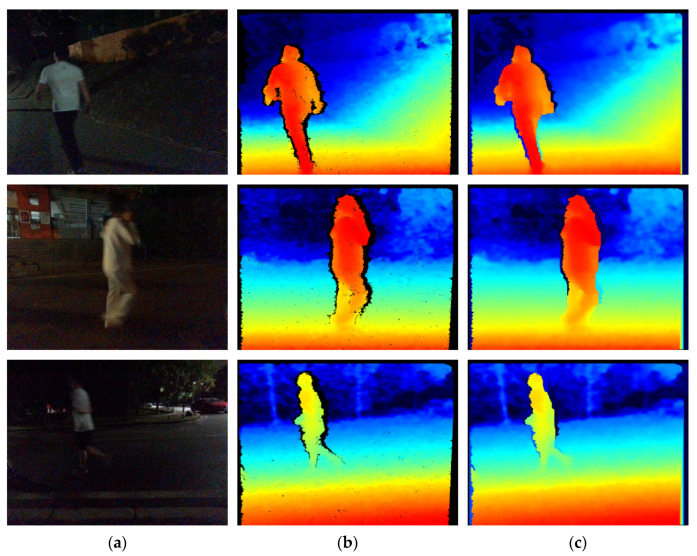
The display of the before and after processing depth images. (**a**) Original RGB images; (**b**) Depth images before processing; (**c**) Depth images after processing.

The corrected RGB images are shown in Figure 2a, and the collected depth images are shown in Figure 2b. RealSense D455 was used as the acquisition device. The processed depth images are shown in Figure 2c. To better cover all kinds of scenes at night, the dataset of this paper contains pedestrian images taken in different scenes and under different lighting conditions on streets and squares at night. The dataset includes 2226 RGB images and their corresponding depth images.

### 3.2. Swin-MFA

**Figure 3:**
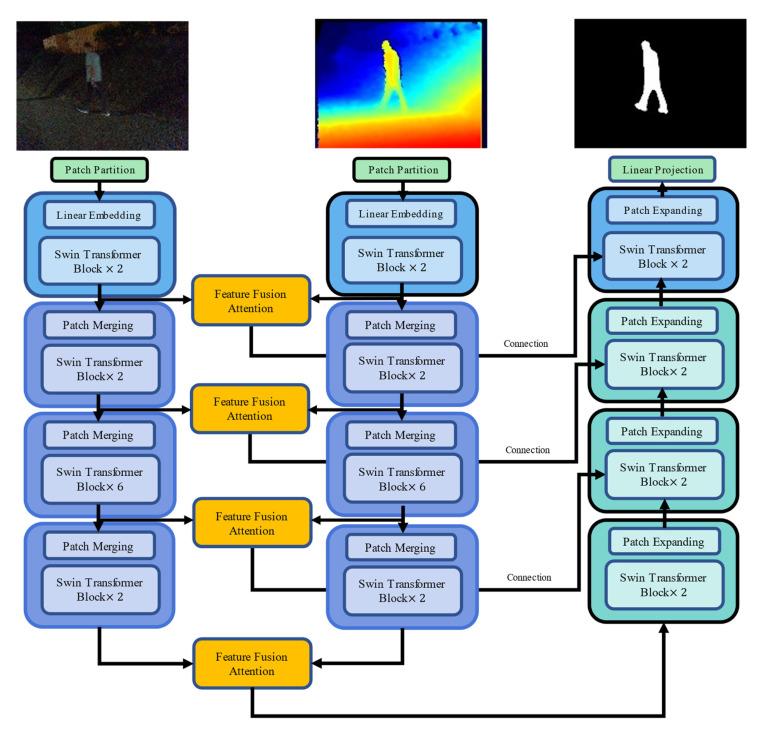
The structure of Swin-MFA network.

Swin-MFA is an improved multi-feature fusion network model based on swin-transformer and Unet structure, which retains the basic structure of the encoder and decoder of Unet. The encoder of the Swin-MFA has two input images, namely the depth image and RGB image. During fusion, the two features, respectively, go through feature fusion mechanism, and the final network features are obtained through the attention calculation and the weighted addition. The decoder uses linear layer amplification and rearrangement for up-sampling, and there are residual connections between the encoder and decoder, which can effectively improve the convergence speed of the network. The specific network structure is shown in Figure 3.

#### 3.2.1. Swin-Transformer Base Backbone Network

**Figure 4:**
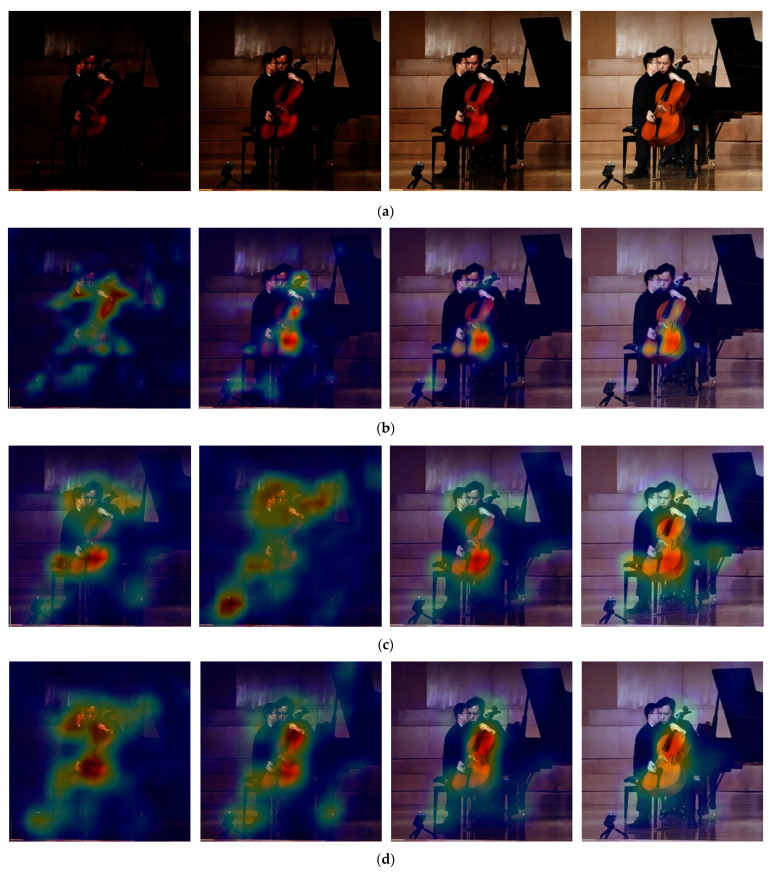

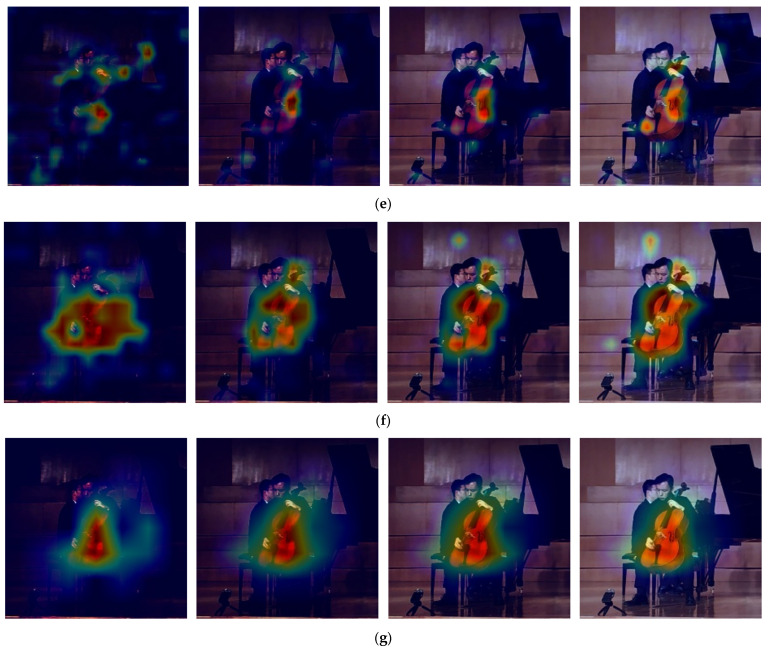
Feature map of different mainstream feature extraction network of cello. (**a**) Original image of different brightness levels; (**b**) MobileNet feature map; (**c**) ResNet-50 feature map; (**d**) ResNet-101 feature map; (**e**) VGG feature map; (**f**) Vision Transformer feature map; (**g**) swin-transformer feature map.

Due to the condition of low illumination, obvious feature information such as color is missing seriously. It leads to the situation, when extracting features, that the backbone network needs to be insensitive to illumination to ensure the reliability in low-illumination conditions. Therefore, it is meaningful to compare the feature extraction structures of mainstream network structures under different lighting conditions in the same scene. Among them, swin-transformer feature extraction layer performs better than Vision Transformer, ResNet, VGG, MobileNet and the encoder of the Unet structure without additional feature extraction layer in the low illumination conditions. Specific experiments are shown in Figure 4.

**Figure 5:**
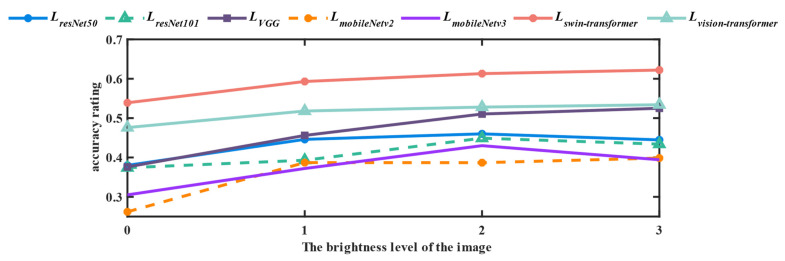
Accuracy diagram of backbone network under different brightness addition.

Using the weights of training on ImageNet, the same image was selected for classification test at different brightness levels, and draw thermal maps by using the grad-CAM [24], which was used to compare the accuracy and concentration of network feature maps at different brightness levels. A score calculation method is defined to measure the feature matching degree of the thermal map on the original image.

(5) $$L=\frac{\sum_{i=1}^{M}\sum_{j=1}^{N}G_{ij}Y_{ij}}{\sum_{i=1}^{M}\sum_{j=1}^{N}G_{ij}}$$

where, *G_ij_* is the two-dimensional output array of grad-CAM. *Y_ij_* is the feature matching area of the original image, and, more specifically, it is −1 when it is background and 1 when it is foreground. The score *L* can be transformed through linear mapping to obtain the result shown in Figure 5.

#### 3.2.2. Self-Attention Mechanism

**Figure 6:**
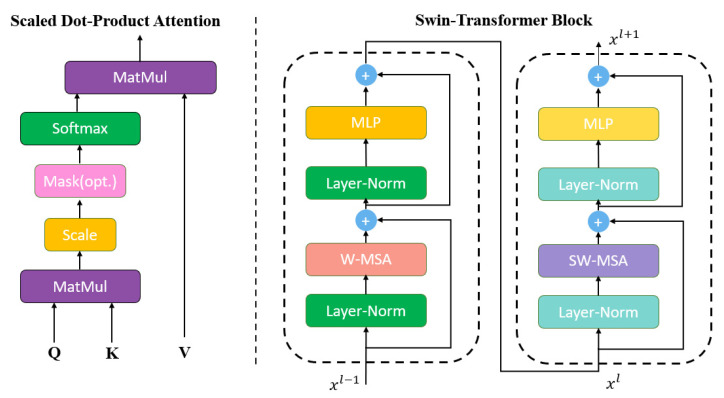
The structure of self-attention mechanism and block in swin-MFA.

**Figure 7:**
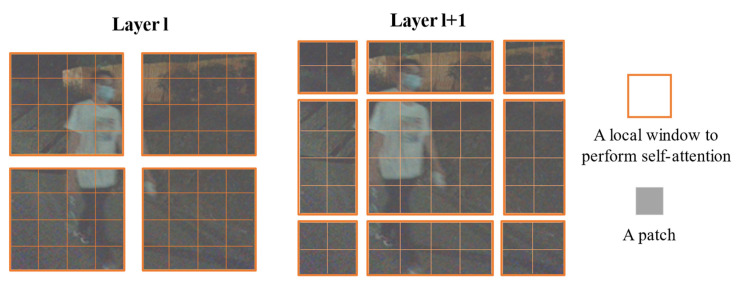
An illustration of the shifted-window approach.

Swin-transformer’s structure contains the form of two multi-headed attention mechanisms, windows multi-head self-attention (W-MSA) and shifted windows multi-head self-attention (SW-MSA). In multi-modal tasks, we also hope to replace the traditional convolutional neural network with the total self-attention. Using the W-MSA module, the network only performs self-attention calculation in windows, and no information is transmitted between windows. Combining with SW-MSA module, the windows slide up to realize information communication between windows, which improves the accuracy and mIoU performance of the network. The specific structure and an illustration of the shifted-window approach are shown in the Figure 6 and Figure 7, respectively.

Moreover, in each swin-transformer block, the sliding-window partition mechanism and calculation can be expressed as the equations in (6).

(6) $$\begin{array}{l} {\hat{x}}^{l}=W-MSA(LN(x^{l-1}))+x^{l-1}\\ x^{l}=MLP(LN({\hat{x}}^{l}))+{\hat{x}}^{l}\\ {\hat{x}}^{l+1}=SW-MSA(LN(x^{l}))+x^{l}\\ x^{l+1}=MLP(LN(x^{l+1}))+{\hat{x}}^{l+1} \end{array}$$

where *W-MSA* and *SW-MSA* are the formula expression of W-MSA and SW-MSA in Figure 5, respectively. The *LN* represents the LayerNorm operation. More precisely, the self-attention mechanism can be a query with a series of key-value pairs mapped to a specific output. It can be expressed by Equation (7).

(7) $$attention(Q,K,V)=softmax(\frac{QK^{T}}{\sqrt{d_{k}}})V$$

where *Q K V* represents three independent matrices, which are the results of different linear transformations of the original sequence *X*, and all of these can be used as representatives of *X*. *d_k_* is the dimension of feature.

#### 3.2.3. Feature-Fusion Attention Mechanism

**Figure 8:**
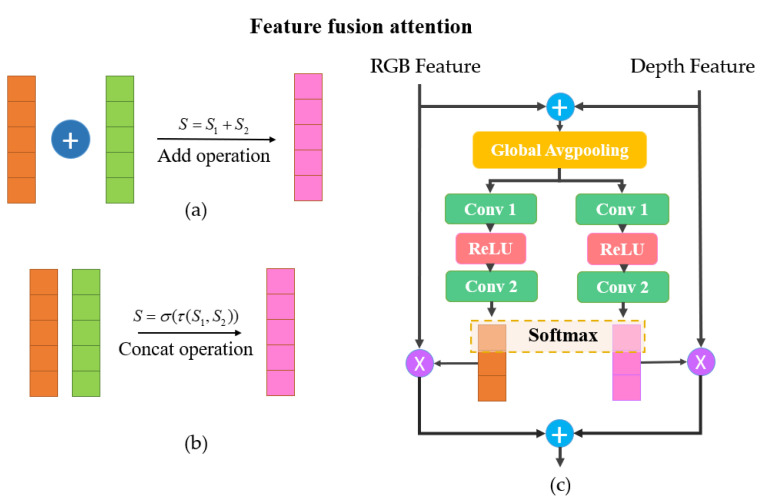
Schematic diagram of image feature fusion operation. (**a**) The schematic diagram of addition operation; (**b**) The schematic diagram of concat operation; (**c**) The schematic diagram of feature-fusion attention mechanism.

For the features of the RGB image and depth image generated in the same scene, there are different processing methods on the feature fusion layer. Inspired by ResNet, an additive operation can be used for the feature fusion. Moreover, concatenate operation is used in DenseNet. For confirming the effect of the feature fusion of the RGB and depth images in low illumination conditions, the experiments on addition operation and concatenate operation are conducted, respectively, which can be identified by Figure 8a,b.

The addition operation increases the amount of information that describes the image, but the feature dimension of the image does not change. With more feature sources contained, the increased amount of information in each dimension is obviously beneficial to image segmentation. However, the concatenate operation is the combination of the channels, in other words, the feature dimension of the image has changed. As the dimension of the concatenate operation is increased, the amount of information under each feature has no essential transformation compared with the addition operation. From another perspective, the addition operation is actually a convolution kernel with the corresponding channels sharing the same weight after concatenate.

Inspired by SKnet [25], we designed a fusion attention mechanism for depth features and RGB features. Through reshaping and the global average pooling of the two features, the convolution kernel activation function operation is used to generate the weight matrix of the two channels, respectively. Moreover, the SoftMax processing is carried out in the horizontal dimension of the features by two independent multi-layer perceptrons and, finally, multiplied with the original input; then, the fusion attention of the two features is realized. More specifically, the structure is shown in Figure 8c, and it can be expressed by the Equations (8)–(11).

(8) $$X_{c}=W_{conv2}\cdot \delta (W_{conv1}\cdot F_{gp}(x_{c}))=W_{conv2}\cdot \delta (W_{conv1}\cdot \frac{1}{H\times W}\sum_{i=1}^{H}\sum_{j=1}^{W}x_{c}(i,j))$$

(9) $$Y_{c}=W_{conv^{\prime }2}\cdot \delta (W_{conv^{\prime }1}\cdot F_{gp}(y_{c}))=W_{conv^{\prime }2}\cdot \delta (W_{conv^{\prime }1}\cdot \frac{1}{H\times W}\sum_{i=1}^{H}\sum_{j=1}^{W}y_{c}(i,j))$$

(10) $$a_{c}=\frac{e^{X_{c}}}{e^{X_{c}}+e^{Y_{c}}},b_{c}=\frac{e^{Y_{c}}}{e^{X_{c}}+e^{Y_{c}}}$$

(11) $$z_{c}=a_{c}\cdot x_{c}+b_{c}\cdot y_{c},c\in [1,C]$$

where *x* and *y* are derived from RGB and depth features with the dimensions H×W×C, respectively. *x_c_* and *y_c_* are the c-th subfeature of *x* and *y*. δ is the ReLU activation function, and $z=[z_{1},z_{2}\cdots z_{C}]$ is the fused attention matrix of the final output. *W_conv_* and *F_gp_* stand for convolution and global pooling operation.

### 3.3. Loss Function

The loss function of the network adopts the cross-entropy loss function, which can be expressed by Equation (12). The mask of the loss function calculation is set to ensure the accuracy of itself. At the same time, Adam is used as the optimizer to train on the LLHS dataset proposed in this paper.

(12) $$Loss(p,q)=-\sum_{i=1}^{C}p_{i}\log(q_{i})$$

where *C* represents the number of categories, *p* is the ground truth and *q* is the predicted result.

## 4. Results

In this section, based on the results from previous experiments in Section 3.2.1, which prove that the swin-transformer backbone maintains relatively stable feature extraction performance in low-illumination scenes, we performed experiments on the LLHS dataset. In Section 4.1, we compare Swin-MFA with various feature-fusion methods, and the experiment proves that the feature-fusion attention block performs better than other traditional methods. In Section 4.2, ablation experiments were performed in the residual connections between the encoder and decoder network. In Section 4.3, we compare our methods with classic image segmentation methods, such as Lraspp Deeplabv3, HRNet, Trans-Unet, and Swin-Unet, as well as with ACNet, RFNet, 3DGNN, ESANet, FuseNet, CEN, etc. RGB-D multi-modal image segmentation methods are also compared. It shows that our network and multi-modal fusion attention mechanism are effective and reliable. In addition, global acc and mIoU, which are commonly used in image segmentation, are also used to evaluate the results. In addition, more specifically, they can be written by Equations (13) and (14).

(13) $$global\;acc=\frac{\sum_{i}n_{ii}}{\sum_{i}N_{i}}$$

(14) $$mean\;IoU=\frac{1}{n_{class}}\sum_{i}\frac{n_{ii}}{N_{i}+\sum_{j}n_{ji}-n_{ii}}$$

where *n_ij_* is the number of pixels with an *i*-th category that is predicted to be the *j*-th categories. *N_i_* is the number of total pixels of the *i*-th category.

### 4.1. Network Fusion Mechanism Experiments

For the fusion mechanism mentioned in Section 3.2.3, we carried out experiments including addition, concatenate with linear cascade and our feature-fusion attention mechanism. The experimental results are shown in Table 1.

It is generally believed that the concatenate operation can cover the addition operations in the effect in improving the segmentation accuracy of the model. However, it is difficult to train the network due to the deep level of the network and the difficulty of convergence. In addition, we notice that the training methods and pre-training weights have an impact on the effect of the feature-fusion methods in the deep network.

### 4.2. Network Connections between Encoder and Decoder Experiments

Considering the connection forms of encoder and decoder and referring to the way of encoder and decoder of Unet, we verified the function of connections between encoder and decoder after the feature-fusion attention mechanism, which are tested in the situations of no connection, single connection and multiple connections, respectively. The specific results are shown in Table 2.

### 4.3. Network Comparative Experiments

**Figure 9:**
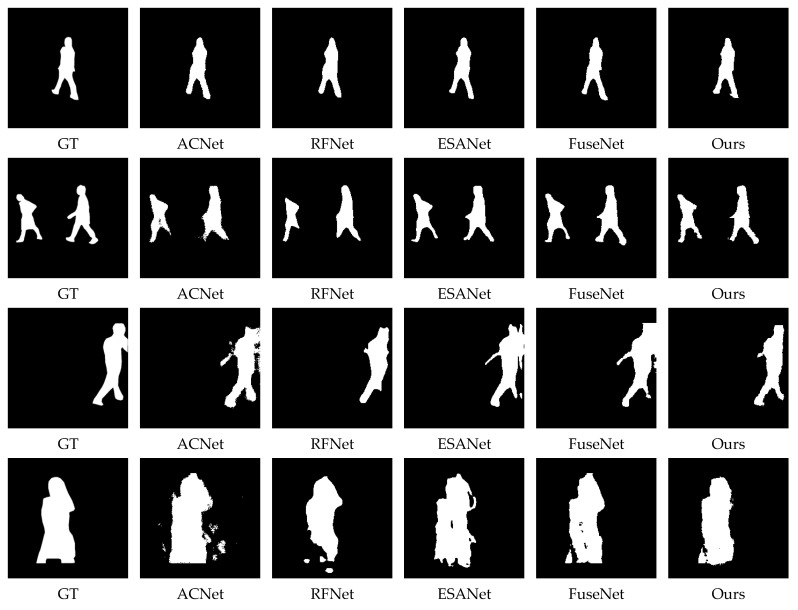

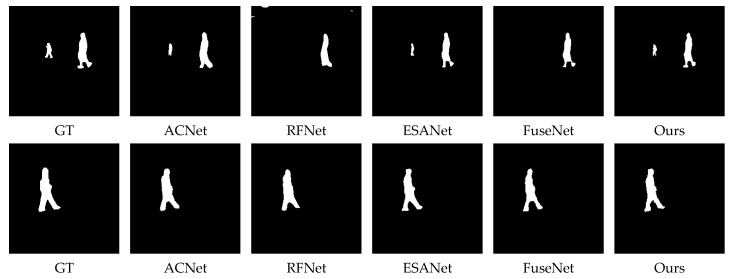
Experimental results compared with other methods.

We compared our methods with Lraspp, Deeplabv3, TransUnet, SwinUnet, ACNet, RFNet, 3DGNN ESANet, FuseNet, LDFNet, etc. The specific results are shown in Table 3 and Figure 9. The experimental results show that our method is effective and accurate.

### 4.4. Experiment of the Combining Datasets of Different Light Intensities

For the low-illuminance monitoring at night, there are occasional lights such as car lights, so it is necessary to conduct data analysis on different brightness conditions. We added five levels of high and low brightness mixing datasets to the LLHS dataset, with high brightness accounting for 10%, 15%, 20%, 25% and 30%, respectively. The specific results are shown in Table 4.

## 5. Discussion

We demonstrate the robustness of a swin-transformer network in low-illumination conditions through comparative experiments and introduce a total self-attention mechanism to replace the traditional convolutional neural network, to improve the ability of the model’s attention to depth images and RGB images. Moreover, a fusion attention mechanism is proposed, to make the overall network have better performance. At present, we are implementing semi-supervised learning on the network, which has made preliminary progress. In the future, we will continue to expand the performance of the network and the active learning ability on datasets without a label.

## 6. Conclusions

In this paper, an end-to-end multi-modal image segmentation transformer network is proposed. Through the multi-modal fusion attention of the depth images and RGB images, the human-segmentation problem in the conditions of low illumination is solved, which can be well-applied in the monitoring and security fields. Depth image and RGB image were used as complementary inputs, and the neural network structure of the multi-modal encoder and decoder was used to realize the segmentation task in complex low-illumination conditions, which improved the robustness and learning performance of the network. In addition, we first propose a low-illuminance human-segmentation dataset, which fills the gap of the multi-modal low-illuminance dataset. Experimental results show that the proposed method is far superior to the advanced single-mode segmentation method as well as the depth and RGB multi-modal network method, with better performance in low-illumination conditions. In the future, we will also realize semi-supervised and unsupervised active-learning strategies by the network, so that the network can still have excellent performance without accurate annotation.

## Figures and Tables

**Table 1:** The results in the experiment of fusion mechanism.

Fusion Method	Global acc	mIoU
Add	93.4	80.7
Concat	92.8	80.8
Ours	93.6	81.0

**Table 2:** The results in the experiment of the number of connections.

Number of Connections	Global acc	mIoU
0	89.2	73.7
1	91.8	76.6
2	92.3	78.8
3	93.4	81.0

**Table 3:** The mIoU and global accuracy results of the comparison experiment.

Method	Global acc	mIoU
Lraspp [26]	73.5	63.4
Deeplabv3 [27]	84.1	54.3
Unet [11]	81.0	71.7
HRNet [28]	46.7	59.1
TransUnet [29]	88.0	75.5
SwinUnet [30]	87.5	69.8
ACNet [23]	92.7	75.7
RFNet [31]	82.4	72.3
3DGNN [22]	92.2	77.6
ESANet [32]	92.2	80.4
FuseNet [33]	84.3	75.3
RedNet [34]	88.5	75.0
LDFNet [21]	89.9	78.3
Swin-MFA (Ours)	**93.4**	**81.0**

**Table 4:** The results of experiment in datasets of different light levels.

Method	10%		15%		20%		25%		30%	
	mIoU	acc	mIoU	acc	mIoU	acc	mIoU	acc	mIoU	acc
ACNet	75.6	92.6	75.0	92.4	75.2	92.0	75.1	92.5	75.1	92.1
RFNet	72.3	82.7	71.5	82.3	71.5	81.8	71.3	82.4	72.1	82.5
3DGNN	77.7	92.5	77.4	92.5	77.1	92.0	77.3	92.1	76.9	91.1
ESANet	80.3	92.2	80.0	92.1	79.8	91.7	80.6	92.2	79.9	91.8
FuseNet	75.1	84.2	74.0	84.4	74.2	84.0	75.5	86.4	74.6	84.7
RedNet	74.8	88.3	71.8	88.4	72.9	87.6	70.6	88.5	73.4	87.4
Swin-MFA	80.8	92.9	80.6	92.9	80.1	92.4	80.5	92.6	80.2	92.9

## Data Availability

The data presented in this study are available on request from the corresponding author. The data are not publicly available due to the privacy issues that the data contained a large number of portraits and pictures of the participants, who did not agree to be posted directly on the Internet. But some applications for research with reasonable requests are still allowed to use.
